# Effects of Apple Consumption on Lipid Profile of Hyperlipidemic and Overweight Men

**Published:** 2011

**Authors:** Mohammad Reza Vafa, Elham Haghighatjoo, Farzad Shidfar, Shirin Afshari, Mahmood Reza Gohari, Amir Ziaee

**Affiliations:** 1 Nutritional Sciences, Associate Professor, President of Iranian Nutrition Society, Iran University of Medical Sciences, Tehran, Iran; 2 Nutrition Department, School of Public Health, Tehran University of Medical Sciences, Tehran, Iran; 3 Statistic Department, School of Management and Medical Information, Tehran University of Medical Sciences, Tehran, Iran; 4 Metabolic Disease Research Centre, Qazvin University of Medical Sciences, Qazvin, Iran

**Keywords:** Malus, Hyperlipidemia, Dietary fiber, Polyphenols, Overweight, Lipid profile

## Abstract

**Objectives:**

Fruits and vegetables may be beneficial on lipid profile of hyperlipidemic subjects. The present study was aimed to verify the effect of golden delicious apple on Lipid Profile in hyperlipidemic and overweight men.

**Methods:**

Forty six hyperlipidemic and overweight men were randomly divided into two groups. Intervention group received 300g golden delicious apple per day for 8 weeks. Control group had the regular dietary regimen for the same period of time. Blood samples were analyzed for serum triglycerides (TG), total cholesterol (TC), low density lipoprotein-cholesterol (LDL-C), high density lipoprotein-cholesterol (HDL-C), very low density lipoprotein-cholesterol (VLDL), apolipoprotein B (Apo B), lipoprotein a (Lp a) and LDL/HDL ratio at baseline and after intervention.

**Results:**

Total polyphenols and fibers were 485 mg/kg and 4.03 g/100g in fresh apple respectively. After 8 weeks, significant statistical differences were observed considering the TG and VLDL levels between two groups, but no significant differences were observed regarding TC, LDL-C, HDL-C, Apo (B), Lp (a) and LDL/HDL ratio.

**Conclusions:**

Consumption of Golden delicious apple may be increased serum TG and VLDL in hyperlipidemic and overweight men. We need more studies to assay the effect of apple consumption on serum TC, LDL-C, HDL-C, Apo (B), Lp (a) and LDL/HDL ratio.

## INTRODUCTION

Epidemiological studies have indicated possible relationships between diet and various chronic diseases, especially coronary heart disease, diabetes mellitus and some malignancies^1^. A fruit and vegetable rich diet (at least 5 servings a day) can reduce the risk by lowering the cholesterol levels.^2^

Positive effects of fruits and vegetables have been attributed to dietary fibers, antioxidants, and especially phenolic compounds.^3^,^4^ Fibers and polyphenols are capable of improving the lipid profile in cardiovascular patients.^5^ Apple is one of those fruits which can play a role in decreasing the risk of chronic diseases, because of the fiber and chemical components such as Flavonoids, Polyphenols and Carotenoids.^6^ Importance of apple can be explained by different factors including their availability in the market throughout the year in a variety of forms (fresh fruit, juice, cider, mashed apples) and also their reputation as a healthy food.^7^ Most studies have focused on how much apple consumption would reduce lipids and their related metabolic disorders.^1^,^3^,^4^,^7^,^16^,^22^ Recent studies have shown the antioxidant properties of apple.^8^ In hyperlipidemia (TG), the increase of total cholesterol (TC), triglycerides, LDL-C, apolipoprotein B and lipoprotein (a) and decrease of HDL-C promotes atherosclerosis and cardiovascular diseases,^9^–^11^ so it seems necessary to develop appropriate strategies in order to regulate lipid profile. The present study was aimed to verify the effect of golden delicious apple on lipid profile in hyperlipidemic and overweight men.

## METHODS

This randomized controlled clinical trial was conducted on 46 hyperlipidemic and overweight men employed in Shahid Rajaii Power Plant in Qazvin, affiliated to the Ministry of Energy, at 2008. Study was approved by the ethics committee of Public Health School of Iran University of medical Sciences and Informed consent was obtained from all participants. the inclusion criteria were as follows; age between 30 to 50, body mass index (BMI) of 25 to 30 kg/m^2^, serum (TC) level of 200-240 mg/dl, serum (TG) of 150-350 mg/dl, no use of antioxidant or lipid lowering agents, no history of diabetes mellitus, thyroid, kidney and liver diseases, hematologic disorders, myocardial infarction, no history of smoking, alcohol or drug consumption. The participants were randomly divided into two intervention and control groups, using table of random digits.

In this study, the 23 individuals of the intervention group received a 300-gram complete raw golden delicious apple daily for 8 weeks. They were recommended not to peel the apples. Control group had the regular dietary pattern, but they were asked not to change their regular diet and physical activity and if possible, to consume fruits other than apple during this period of time. The intervention group received their daily apple once a week in packs of 300 g (seven packs of 300 g) at the power plant and consumed it as a snack at 10 am and 4 pm. In this study, only raw unpeeled apples were used and cooked or canned apples were not used. People who, for any reason, could not consume raw unpeeled apples were not recruited in the study.

Before the study, the anthropometric data, including height [without shoes, in centimeter (cm), using Rasa stadiometer] and weight [without shoes, with the minimal clothing, in kilogram (kg), using Seca scale] were measured and BMI was calculated. The International Physical Activity Questionnaire^12^ and the validated Food Frequency Questionnaire (FFQ), were used to check the usual amount of fruit and vegetable received daily by the participants. A 24-hour food recall questionnaire were completed by both intervention and control groups, in the three first days of the study (an off day and two working days) and the three last days of the study. The 24-hour food recall questionnaires were then analyzed, using Nutritionist4 software and the total calorie, carbohydrate, lipid, protein, fiber, antioxidant vitamins (A, C, E) and cholesterol intake was calculated.

### 

#### Laboratory Evaluations :

After fasting for 12-14 hours, a 10 ml venous blood sample was taken from the left arm before and after the study. Concentration of Triglyceride, Total Cholesterol, LDL-C, and HDL-C were measured by an enzymatic method, using “Pars Azmun” commercial kits (manufactured in Tehran, Iran, under the license of German Herb Company). Through this method TG was measured with a sensitivity of 5 mg/dl and accuracy of 1.6%, TC with a sensitivity of 5 mg/dl and accuracy of 1%, LDL and HDL, each with a sensitivity of 1 mg/dl and accuracy of 0.65% and 0.81% respectively Lp (a) with a sensitivity of 2 mg/dl and accuracy of 3.4%, through Enzyme Linked Immunosorbent Assay (ELISA) method, using Neuvillesur-Oise, Hyphen Bio-Med, France commercial kit; Apo (B) with a sensitivity of 0.3 mg/dl and accuracy of 4.1%, through ELISA method, using ALerChek, Portland commercial kit And VLDL was calculated by dividing the Triglyceride value by 5 (Friedewald formula).^13^

#### Statistical methods:

The student t-test was used to compare the quantitative variables between the two groups and the paired t-test was used for comparing the ranges of differences in each group. In order to evaluate the relationship between qualitative variables, Chi Square test was performed. Data was analyzed using SPSS version 16. A p-value under 0.05 was considered to be significant.

#### Compounds in apple:

Among compounds found in apples, Total Polyphenolic and Total Fiber compounds were measured. The Polyphenol was measured with High Performance Liquid Chromatography (HPLC), through W. Plocharski and J. Markowski method^14^ and the total fiber was measured through American Oil Chemists Society (AOCS) method.^15^

For HPLC which was carried out with a sensitivity of 0.1 part per million (ppm) and accuracy of 0.5 ppm, 10 g of ground up apples were homogenized for 1 minute with 70% aqueous ethanol solution. The result suspension was transferred to a 50 ml flask which was filled to the mark with the 70% methanol. The result mixture was then filtered through whatman No. 1 filter paper. The filtrate was stored at -18°C prior to analysis. Before HPLC, the samples were diluted at the ratio of 1/3, by sodium acetate buffer (solution A). HPLC was carried out, using Young Lin Acme 9001 system equipped with a DAD detector. Polyphenols were separated using Lichrosphere C18 RP-100. The mobile phase consisted of 10.2% acetic acid in 2mM sodium acetate (solution A) and acetonitrile (solution B). The flow rate was kept constant at 0.5 ml/min, for the total time of 72 minutes at 25° C. The system was separated with a gradient program 3% B (0-20 min), 3- 35%B (45 min), 35- 90% B (3 min), 9-90% B (4 minutes), 90- 0% B (1 min). The column was equilibrated in 10 minutes in initial conditions. The material volume injected into the column was 20 μl.

In order to measure the Total Fiber, 0.2 g of unpeeled apple was refluxed with 200 ml normal sulphuric acid for 1 hour. The refluxed sample was filtered and then refluxed by 200 ml of 313% normal soda and filtered. Filter paper was placed in an oven for 15 minutes. The percentage of crude fiber was calculated by the following formula:

$$\mathrm{Crude}\;\mathrm{fiber}\;\mathrm{percentage}=\frac{weight\;of\;\sec ond\;filter\;paper–weight\;of\;first\;filter\;paper}{Sample\;weight}\times \left(humidity-100\right)$$

To calculate the humidity, 5 g of ground up sample was poured in a plate and placed in an oven for an hour.

$$\mathrm{Percentage}\;\mathrm{of}\;\mathrm{humidity}=\frac{\sec ond\;plate\;-f\mathrm{irst}\;\mathrm{plate}\;\times \;100}{\mathrm{Sample}\;\mathrm{weight}}$$

The concentration of Polyphenolic and Fiber compounds was 485 mg/ kg fresh weight and 4.03% (4 g per 100 g fresh apple) respectively; the value of Polyphenol and Fiber received daily by the intervention group from a 300-g golden delicious apple was 145.5 mg and 12 g respectively.

## RESULTS

There was no significant statistical difference between the two groups regarding age, BMI, education and family size. Changes in physical activity was the same in both groups, no significant difference was seen between the two groups considering physical activities before and after the study. Table 1 shows the baseline characteristics of the participants.

**Table 1:** Characteristics of intervention and control group before the study

	Intervention group	Control group
Number	23	23
Age*	41.08±4.19	41.65±3.79
Weight (kg)	80.04±5.53	79.82±8.69
IBM (kg/m^2^)	27.02±1.39	26.72±1.83
Education: ¶		
No diploma received	2 (8.7)	3 (13)
Diploma	9 (39.1)	8 (34.8)
A.A.#	6 (26.1)	3 (13)
B.A.¥	6 (26.1)	9 (39.1)
Number of people in household:		
2	1 (4.3)	2 (8.7)
3	8 (34.8)	7 (30.4)
4	8 (34.8)	11 (47.8)
5	4 (17.4)	3 (13)
More than 5 Physical activity:		
Light	3 (13)	10 (43.5)
Moderate	16 (69.6)	6 (26.1)
Severe	4 (17.4)	7 (30.4)

* Data listed in the table above are shown based on (mean ± standard deviation)

¶ Data listed in the table above are shown based on Numbers and (Percent)

# Upper Diploma

¥ Bachelor of Science

The mean level of vitamin A and vitamin E intake, showed a significant statistical difference among two groups; the intervention group showed a significant increase in each gram fruit intake (p= 0.0001) compared the control group. In other cases, no significant difference was observed between the two groups.

After the study, the mean level of TG and VLDL concentration showed a significant decrease in control group compared to the intervention group (p= 0.04 and p= 0.001 respectively). However, the increase of TG and VLDL concentration observed in intervention group was not significant compared to the concentration measured before the study. Serum level of TC, LDL-C, HDL-C, Lp (a), Apo (B) and LDL/HDL ratio had no significant difference between the two groups. It should be noted that among atherogenic factors, only the decrease of Lp (a) was higher in intervention group than control group which was not statistically significant (p=0.055). Table 2 shows the mean level of lipid profile in the study groups.

**Table 2:** Lipid profile in two groups before and after study

Biochemical parameters	group	Before study	After study	Mean differences
TG (mg/dl)	Intervention	216.69±52.59	224.21±84.6	7.52±32.01^a^
	Control	219.52±67.54	183.17±53.38	-6.35±14.16^a^
TC (mg/dl)	Intervention	220.13±13.47	213.43±18.76	-6.7±5.29
	Control	216.82±14.51	209.78±21.18	-7.04±6.67
LDL-c (mg/dl)	Intervention	130.69±13.28	125.34±13.59	-5.35±0.31
	Control	127.95±11.53	121.26±16.6	-6.69±5.07
HDL-c (mg/dl)	Intervention	42.13±7.05	39.6±6.2	-2.53±0.85
	Control	43.3±8.29	41.56±8.92	-1.74±0.63
VLDL (mg/dl)	Intervention	44.69±13.23	44.82±16.95	0.13±3.72^b^
	Control	43.86±13.45	36.43±10.86	-7.43±2.59^b^
LDL/HDL	Intervention	3.15±0.63	3.13±0.63	-0.02
	Control	2.98±0.65	2.97±0.64	-0.01±0.01
Lp(a) (mg/dl)	Intervention	27.39±16.09	20±9.48	-7.39±6.61
	Control	23±13.23	19.34±8.98	-3.36±4.25
Apo(B) (mg/dl)	Intervention	130.04±13.58	131.3±10.43	1.26±3.15
	Control	126.47±13.88	125.17±13.03	-1.3±0.85

Data listed in the table above are shown based on (mean ± standard deviation)

a. The result of independent t-test shows that the mean level of differences in TG concentration has a significant decrease (p=0.01) among control group in relation to the intervention group

b. The result of independent t-test shows that the mean level of differences in VLDL concentration has a significant decrease (p=0.04) among control group in relation to the intervention group

## DISCUSSION

The results showed that apple consumption had no significant effect on lipid reduction in overweight men which agrees with some other studies^16^–^18^. in a study by Hyson et al. which was conducted on 28 subjects with normal cholesterol level, by consuming 375 ml apple juice or complete 340gr apple for 6 weeks, no significant difference was observed in serum levels of TC, LDL-C, HDL-C and Apo (B) and a low, insignificant increase was observed in fasting TG level.^16^ In another study by Oliveira et al. which was performed on 49 hypercholesterolemic women, daily consumption of 300 gr for 12 weeks increased significantly the TG concentration and decreased insignificantly the TC level.^17^

In a study by AVC et al. which was conducted on 15 aged people with the average age of 67-75, after receiving 2 gr. apple per kg body weight daily for one month, no significant difference observed in the level of serum TG, TC, LDL, HDL and VLDL.^18^

In a study by Dennison et al., consumption of 5.5 ounces (158.7 g) fruit juice including a mixture of 35% (1.8 ounces) apple juice, 31% (1.5 ounces) orange juice, 25% (1.3 ounces) grape juice and 9% other types of fruit juices caused no significant difference on the level of TG, TC, LDL-C and Lp (a).^19^ In a study by Davidson et al. on 110 hypercholesterolemic subjects, by consuming 720 ml apple juice together with gum Arabic and Pectin (by a ratio of 4/1), for a washout period of 12 and then 6 weeks, in which the participant received 720 ml mere apple juice, the mean serum level of TC and TG increased by 3.5% and 28.5% respectively during the 12 weeks, while HDL and LDL levels showed no significant difference. During washout period, TC mean level had a significant increase of 2.4%. Finally, the hypothesis of serum cholesterol reduction by apple juice consumption was rejected in this study.^20^

The mechanism which would result in an increase of serum TG in the above studies and the present one may be an increased intake of fructose by apple consumption. An augmentation of dietary fructose intake may increase TG due to the increase of the liver lipogense enzymes activity.^20^ On the other hand, although VLDL concentration showed a significant decrease among control group and an insignificant increase among intervention group; since the increase in Apo (B) concentration was not significant among intervention group, the number of Apo (B) particles did not differ and the number of VLDL particles decreased and for this reason, these particles are larger and lighter and they have less ability to penetrate vessel walls. Therefore, there would be a lower atherogenic risk associated to these particles among intervention group.^21^ Furthermore, since the absorption of biological active compounds present in a full meal may be decreased under the effect of such variables as chewing,^16^ the insignificant effect of apple consumption on lipid profile in the present study may be related to the decrease in the absorption of active components present in apples.

Results form some studies disparate from those in the present study. in a study by Nagasako et al., a daily intake of 600 mg apple polyphenol extract caused a significant decrease of serum TC and LDL-C^22^ a 300-gram golden apple used in Nagasako study contain 145.5 polyphenol, so the 600 polyphenol extract equals 1200 g golden apple, which is 4 times more than the amount that has been used in our study. Therefore, since in our study, first a complete apple fruit has been used and the effect of compounds present in an apple is less than the effect of purified compound in the supplement, more time is needed to observe the effect of its ingredients. Second, the daily intake of polyphenols was lower in our study than in the Nagasako study (145.5 mg/day in comparison with 600 mg/day), we may attribute the insignificant result of this study to the short duration and the low amount of polyphenol present in the type of apple consumed.

Besides that, since the subjects of the intervention group have been selected from the personnel of Shahid Rajaii Power Planet who are examined every year, considering the “Healthy Worker Effect Phenomenon”, they are healthier than the general population.^23^ Therefore; daily intake of apple with low polyphenol cannot have positive and significant effect on the level of lipid profile. While in similar studies whereby significant effect has been observed, the target society has generally been selected from the general population.

In the present study, the polyphenol present in golden delicious apple, red delicious apple and granny smith apple was calculated 485, 620 and 835 ml/ kg fresh apple fruit weight^14^ respectively; and the polyphenol received daily by the intervention group from a 300-gram golden delicious apple was less than red delicious apple and granny smith apple by 1.3 and 1.7 times respectively. This can be another reason why the lipid profile did not show any significant decrease compared to similar studies.

On the other hand, in a complete fruit, fiber and phenolic compounds have great synergy effects,^7^ but in this study, despite the presence of high fiber (4 g in 100 g fresh apple fruit), due to the shortage of polyphenols value, fibers had no notable effect on cholesterol reduction. Furthermore, since after the study, fiber intake among intervention group showed no significant difference compared to the control group, this can explain why the fiber present in the apple had no effect on serum lipid reduction.

Indeed, the general expectation from higher consumption of apples among intervention group was to observe a significant reduction in the level of lipid profile due to increase in fiber intake and other antioxidant components present in apples; however this result was not observed in this study. Also due to operational reasons, it was not possible to use placebo in control group, and the educational effect of the present study may be the result of behavior change in control group which can have a positive effect on their pathological and Para clinic results and finally cause an insignificant effect of apple consumption.

In this study, the golden delicious apple increased the serum levels of VLDL and TG but had no effect on TC, LDL-C, HDL-C, LDL/HDL ratio, Lp (a) and Apo (B) which can be due to the increase of fructose intake, the low value of polyphenol in the type of apple used and thereby the diminish of fiber and polyphenol synergy, the low number of subjects and insufficient duration of the study.
